# Leveraging Community‐Based System Dynamics to Understand Long Covid Disparities in African American Communities: A Model for Health Equity Research

**DOI:** 10.1111/hex.70516

**Published:** 2025-12-15

**Authors:** Chad R. Evans, Melvin R. Echols, D. Martin, H. A. Taylor, James A. Washington, Olusola Gbinigie, Anne H. Gaglioti, Wendi Wright, Peter Hovmand

**Affiliations:** ^1^ Morehouse School of Medicine Cardiovascular Research Institute Atlanta Georgia USA; ^2^ Global Institute for the Learning Society Oakland California USA; ^3^ Morehouse School of Medicine Clinical Research Center Atlanta Georgia USA; ^4^ American Cancer Society Atlanta Georgia USA; ^5^ Case Western Reserve University Cleveland Ohio USA

**Keywords:** causal loop diagram, Community‐Based System Dynamics, group model building, health equity, long Covid, participatory research, patient engagement

## Abstract

**Background:**

Long Covid disproportionately affects African American communities, exacerbating pre‐existing health disparities and systemic barriers to care. Conventional public health interventions often fail to address the complex systemic issues at play due to a lack of grounding in community‐certified knowledge about the broader societal context that produces the disparities and in which the interventions must operate. New methods are needed to elicit community perspectives on living with long Covid.

**Methods:**

This study employed Community‐Based System Dynamics (CBSD) workshops, conducted in hybrid formats (online and in‐person), to engage African American communities impacted by long Covid. Participants included affected individuals, healthcare professionals and systems researchers.

**Results:**

The workshops yielded system dynamics causal loop diagrams that illustrate the multifaceted societal context and impact of long Covid. Community‐driven insights led to the identification of targeted interventions and informed a comprehensive action plan designed to address specific health system barriers and enhance community resilience.

**Conclusions:**

CBSD workshops proved effective in fostering significant community engagement and empowerment, presenting a replicable model for gaining a deeper understanding of the socio‐cultural context that underlies complex health disparities. These findings suggest that incorporating community‐sourced societal context knowledge and system dynamics modelling and analysis can substantially enhance public health strategies for managing long Covid.

**Patient or Public Contribution:**

People with lived experience of long Covid were actively involved throughout all phases of this study. Participants contributed to the design and facilitation of Community‐Based System Dynamics (CBSD) workshops, helped construct and refine causal loop diagrams based on their experiences, and generated action ideas for future interventions. Their insights shaped both the structure and content of the system models and directly informed the interpretation of results. Several participants also reviewed and provided feedback on early drafts of the manuscript to ensure the findings reflected their perspectives and priorities.

## 1 Introduction

Post‐acute sequelae of SARS‐CoV‐2 infection (PASC), also known as long Covid, persists as a significant aftermath of the Covid‐19 pandemic, characterised by a range of symptoms that continue long after the initial infection has resolved and defined as persistent, relapsing or new symptoms and conditions that present 30 or more days after Covid‐19 infection [1]. African American communities face a disproportionately high burden of these ongoing symptoms, compounding long‐standing health inequities [2, 3]. A growing body of evidence from large‐scale epidemiological studies, national surveys and meta‐analyses has revealed a clear and troubling trend: Black and Hispanic communities bear a disproportionately high burden of long Covid. Major cohort studies and federal data analyses consistently show that these populations experience a higher prevalence and severity of long Covid, and these disparities remain robust even after adjusting for socio‐economic and clinical factors [4, 5, 6]. This inequity has been documented across diverse settings, from large urban cohorts to national electronic health records, and has been a key finding of the national RECOVER initiative [7, 8, 9]. The traditional public health response, which tends to be compartmentalised and reactive, may not fully address the complexities of long Covid [3]. This situation calls for innovative approaches that recognise and address the systemic nature of health disparities and are grounded in community‐sourced societal context knowledge [10].

Recognising the limitations, we advocate for Community‐Based System Dynamics (CBSD) as a transformative approach to health research. CBSD is a participatory approach that engages community members as experts in their own lived experience, empowering them to collaboratively map the socio‐structural factors and feedback loops that influence their health [11]. By co‐creating system maps, CBSD not only ensures that research is contextually relevant but also builds community capacity and identifies leverage points for effective, sustainable interventions [12, 13]. To our knowledge, this is the first study to explicitly use CBSD to understand the systemic challenges of long Covid within the African American community.

This study harnessed the insights of African American individuals directly impacted by long Covid through a series of CBSD workshops [14]. By integrating their lived experiences with system science principles, we aimed to develop a shared, systemic understanding of managing long Covid, identify key feedback loops that perpetuate disparities, and lay the groundwork for community‐vetted strategies to promote health equity. This paper presents the findings generated through this participatory process and discusses the broader implications of CBSD for public health practice and policy in a post‐pandemic world.

## 2 Methods

### 2.1 Study Design

The design process for the three CBSD workshops combined in‐person and online formats to accommodate participant needs and maximise the reach of engagement, facilitating an agile and responsive approach to model development [15]. Serving as a central component of the study design, the CBSD workshops employed a structured yet participatory approach to integrate lived experience directly into the development of system models depicting the complex dynamics of living with long Covid. Adapting methods like group model building (GMB) to online settings has been explored as a complementary option to face‐to‐face delivery, particularly when restrictions limit in‐person activities [16]. While online formats can present challenges regarding access and diversity of participants, they can also potentially facilitate broader inclusion by reaching individuals who might otherwise be unable to attend [15]. Flexible delivery is recognised as an implementation factor that can influence the success of systems thinking approaches in community settings.

The first in‐person workshop convened researchers from an existing long Covid study, participants enrolled in the study with lived experience, and CBSD experts over a period of 2 days in January. CBSD and GMB often involve diverse stakeholders, including community members, researchers and experts [17]. Initial workshops in the development of a causal loop diagram (CLD) typically focus on problem scoping and identifying key variables and the system boundary [18]. The primary objective of this foundational session was to collaboratively explore participant experiences with long Covid through structured GMB activities, engaging participants in initial CLD exercises aimed at identifying key system dynamics, relationships among symptoms, and contextual factors shaping the condition's trajectory [16].

We conducted a follow‐up online session with participants from the in‐person workshop that was conducted on 16 February 2024. This session provided an opportunity to revisit and refine the emerging model, validate thematic structures, and introduce additional variables and feedback loops based on participant reflection. A third and final workshop was held online on 23 April 2024 and included a new group of participants with lived experience of long Covid, specifically those whose symptoms, location or circumstances precluded in‐person attendance. This session followed a similar structure but emphasised action idea generation and participant feedback on an exploratory simulation model derived from earlier workshops. The exploratory model was from a previous CBSD workshop on a different topic (cardiovascular health) and a different study and used to introduce and explore interest in developing a similar simulation model from this workshop. The use of such exploratory models being shared across problem contexts within a community (e.g., same community, different topic and patient groups) is an effective way to build a community‐based practice in system dynamics that is specific to CBSD. We make the simulation model, along with documented equations, available at GitHub public repository (https://github.com/CBSDLab/GSPA). Collectively, this mixed‐format design allowed for iterative model development, ensuring that both the rich dynamics of in‐person interaction and the enhanced accessibility of online engagement contributed to a more comprehensive systems understanding of long Covid, reflecting responsiveness to community needs and facilitating broader inclusion.

### 2.2 Workshop Outline

Each workshop followed a structured agenda of facilitated activities (or ‘scripts’), which began with rapport‐building exercises before progressing to core system mapping tasks (see Table 1 for a detailed outline).

**Table 1:** Summary/outline of workshop activities.

Workshop activity	Description
Welcome and overarching goals	The initial phase of the workshop focused on introducing participants, building rapport and clearly communicating the purpose, objectives and overall process of the sessions.
‘Hopes and Fears’ for this workshop	A participatory exercise where individuals share their expectations, motivations, concerns and desired outcomes regarding their involvement in the workshop process.
Behaviour over time graphs related to long Covid symptoms and treatment (in‐person only)	A method used to graphically plot the historical patterns or perceived trends of key variables (e.g., symptom severity and treatment access) over time to identify dynamic behaviours.
Causal loop diagramming (developing causal loop diagrams)	The core process of collaboratively identifying key variables within a system and mapping the perceived cause‐and‐effect relationships between them, forming feedback loops.
Action ideas (online only)	A brainstorming session dedicated to generating potential interventions, policies or strategies aimed at addressing challenges and improving outcomes based on the system maps.

### 2.3 Participant Recruitment

All in‐person workshop participants were randomly recruited from an existing 12‐month observational study (*n* = 80) investigating long Covid among patients at Grady Memorial Hospital (GMH), a 953‐bed Level 1 trauma centre serving the greater Atlanta metropolitan area. The original observational study utilised a combination of telemonitoring and smartphone‐enabled technology to monitor clinical and objective symptoms following Covid‐19 infection (see Table 2) [19]. While the study was not powered to test the hypotheses associating observed data with prolonged Covid‐19 symptoms, we examined the distribution of patient characteristics, estimates of effect and variances of measurements to inform a future clinical trial [20].

**Table 2:** Demographic and clinical characteristics of the original observational cohort and the workshop participant sub‐sample (Demographic and clinical characteristics of study participants: Original versus workshop groups presents the demographic and clinical characteristics of the full observational study sample [*N* = 80] compared to the subset who participated in the CBSD workshops [*N* = 29]).

Characteristic	Original sample (*N* = 80)	Workshop sample (*N* = 29)
Age, mean (SD), years	41.2 (18.7)	67.4 (13.7)
Gender, *n* (%)		
Female	44 (55.0)	18 (62.1)
Male	36 (45.0)	11 (37.9)
Risk factors for long Covid (PMH of: obesity, diabetes, hypertension, asthma, autoimmune conditions, HIV or immune suppression), *n* (%)		
Present	38 (47.5)	25 (86.2)
Absent	42 (52.5)	4 (13.8)
Covid‐19 vaccination status, *n* (%)		
Vaccinated	51 (63.7)	20 (69.0)
Unvaccinated	29 (36.2)	9 (31.0)
Breakthrough infection, *n* (%)		
Yes	16 (20.0)	2 (6.9)
No	64 (80.0)	27 (93.1)
Insurance coverage, *n* (%)		
Yes	57 (71.2)	17 (58.6)
No	23 (28.7)	12 (41.4)
6‐min walk test completion, *n* (%)		
Completed	63 (78.8)	21 (72.4)
Not completed	17 (21.2)	8 (27.6)
Vital status, *n* (%)		
Alive	73 (91.2)	29 (100.0)
Deceased	7 (8.8)	0 (0.0)
ED/clinic visits after Covid‐19 infection, mean (SD)	2.28 (1.73)	4.82 (3.96)
Workshop location, *n* (%)		
In‐Person	—	15 (51.7)
Online	—	14 (48.3)

*Note:* The ‘Workshop Sample’ column (*N* = 29) includes data only for participants recruited from the original observational study. An additional 5 patient advocates, for whom prior clinical data were not available, also participated in the CBSD workshops, bringing the total number of workshop participants to 34.

A total of 34 individuals participated in the CBSD workshops. These participants included 29 individuals recruited from the original observational study cohort and 5 external patient advocates living with long Covid. All 34 individuals contributed to the qualitative group model‐building activities. Because detailed demographic and clinical data were collected as part of the parent observational study, the quantitative characteristics presented in Table 2 are limited to the 29 participants recruited from that cohort. All participants in both the original observational study cohort and the CBSD workshops identified as African American [21].

The observational study and the subsequent CBSD workshops were approved by the Morehouse School of Medicine Institutional Review Board. We obtained written informed consent from all participants before their enrollment in the original observational study, and separate informed consent was obtained from all 29 participants who agreed to participate in the CBSD workshops after receiving a full explanation of the workshop's purpose and procedures.

## 3 Results

The in‐person CBSD workshops yielded a comprehensive CLD and participant insights regarding long Covid [22, 23]. The initial in‐person workshop resulted in the collaborative development of behaviour over time graphs illustrating the perceived trajectories of symptoms and experiences with treatment, alongside preliminary CLDs mapping key relationships. Participants in this session particularly highlighted the central role of themes such as trust in healthcare providers, validation of their lived experiences, and the significant interplay between symptom severity and social functioning [24].

Given the nature of certain long Covid symptoms, such as severe fatigue, cognitive dysfunction, anxiety and depression, which can impede attendance at in‐person workshops, the study team elected to conduct an online CBSD workshop [25]. Online workshops facilitated broader geographic reach, offered scheduling flexibility and enhanced accessibility, rendering them a viable option for geographically dispersed participants or those experiencing symptoms that impede their ability to leave the house [26]. The online workshops expanded the scope and detail of the system model built during the in‐person workshop. Participants refined the initial causal structures, introducing additional variables and feedback loops to represent a broader range of influencing factors.

Collectively, the iterative process across the in‐person and online workshops produced a co‐developed causal loop model representing the complex system of factors influencing long Covid, grounded in the lived experiences of affected individuals [27]. This process also generated qualitative data on participants' hopes, fears and potential solutions. These outcomes demonstrate the capacity of a mixed‐format CBSD approach to generate detailed system insights and actionable ideas, particularly valuable when working with populations affected by conditions that impact participation [21, 28].

## 4 Hopes and Fears Exercise

### 4.1 In‐Person

Participants expressed a strong desire to discuss their hopes and fears related to living with long Covid, rather than focusing solely on the anticipated outcomes of the workshop. This unexpected focus revealed the profound psychological and social impact of long Covid on individuals' lives. Interestingly, the depth and richness of these responses were closely aligned with current research on social determinants of health (SDoH), highlighting the intersection of chronic illness with broader socio‐economic and environmental factors [29]. This deviation underscores the importance of addressing the holistic needs of long Covid patients, beyond clinical symptoms, to encompass their emotional and social well‐being [30]. Table 3a summarises the In‐person Hopes and Fears exercise results.

**Table 3a:** Summary of in‐person participant hopes and fears regarding long Covid (Table presents the hopes and fears regarding long Covid articulated by participants during the in‐person CBSD workshop. Entries are categorised by the overarching thematic area based on the summary of expressed sentiments under hopes or fears).

**Session**	**Hopes**	**Fears**
In‐Person	Scientific advancements Hope for a cure for long Covid or management of long‐term effects Hope for better research outcomes and new natural remedies Hope to apply research to broader topics	Health deterioration Fear of death and worsening health Fear of medication side effects and persistent symptoms like shortness of breath Fear of cognitive decline, especially memory loss
	Medical and professional improvements Hope for more compassionate doctors Hope for comprehensive training for medical professionals about chronic conditions	Systemic barriers Fear of treatments not being affordable or accessible Fear that research results will arrive too late Fear that research outcomes will not reach the average person
	Community and support Hope to meet and support others in the community Hope for self‐protection and mutual aid Hope for acknowledgement of the seriousness of the condition	Loss of credibility and knowledge Fear that the knowledge gained will not be credible or helpful Fear of being unable to help others effectively
	Broader societal hopes Hope to prevent societal shutdowns and help vulnerable populations	Social isolation Fear of returning to social isolation

### 4.2 Online

The online workshop offered increased flexibility in how we conducted the hopes and fears exercise. The online format allowed for the establishment of five virtual breakout groups. The participants for each breakout group were selected randomly. In the breakout groups, participants were allocated 10 min to discuss their hopes and fears related to living with long Covid. This exercise aimed to elicit personal reflections and collective insights on the challenges and aspirations associated with the condition. Following these discussions, each group was asked to share a brief summary of its conversations with the larger group. This summary included the key themes and concerns identified during their discussions, providing a broader understanding of the lived experiences and emotional landscapes of those affected by long Covid [26]. Table 3b summarises the online Hopes and Fears exercise results.

**Table 3b:** Summary of online participant hopes and fears regarding long Covid (Table presents the hopes and fears regarding long COVID articulated by participants during the online CBSD workshops. Entries are categorised by the overarching thematic area based on the summary of expressed sentiments under hopes or fears).

**Session**	**Hopes**	**Fears**
Online	Access to care Hope that health insurance will provide sustained support for treatment and diagnosis Hope for the establishment of clinics specialised in long Covid care	Health outcomes Fear that symptoms will worsen over time Fear of continuous symptom management, such as wear and tear Fear of persistent symptoms, anxiety and functional decline
	Treatment approach Hope for non‐pharmaceutical treatment options	Infection risk Fear of getting sick again due to inadequate public health behaviours Fear of reinfection
	Health outcomes Hope to avoid infection	Misinformation Fear of misinformation and conspiracy theories
	Community support Hope for a supportive community for self‐care	Developmental concerns Fear of developmental impacts on children born during the pandemic
	Spiritual and emotional support Hope for maintaining spiritual connection during recovery	Access to benefits Fear of losing disability benefits over time
	Treatment development Hope for the development of effective treatments	Uncertainty about the future Fear of ongoing uncertainty and absence of clear solutions
	Recognition and understanding Hope for increased recognition, understanding and knowledge of long Covid	Advocacy and representation Fear of being unable to advocate for one's self during critical healthcare moments
	Research progress Hope that research will enable better future outcomes for the next generation Hope that current research is progressing towards a solution	Family responsibilities Fear associated with raising children while managing illness
		Symptom confusion Fear of difficulty distinguishing Covid symptoms from other illnesses
		Diagnostic challenges Fear of receiving inaccurate diagnoses or being misclassified
		Health equity and stigma Fear of being stigmatised through existing health disparities Fear of being labelled as a hypochondriac

## 5 Behaviour Over Time Graphs

During the in‐person session, participants were tasked with hand‐drawing behaviour over time graphs. The graphs focused on symptoms and treatments related to long Covid, providing a detailed visual representation of the progression and management of this condition. These graphs illustrated trends in symptom severity, treatment efficacy and patient outcomes. Additionally, the discussions extended beyond the immediate medical aspects, encompassing broader social issues such as homelessness and the challenges faced by individuals living with Covid. The implications for nurse employment were also a significant topic, highlighting the strain on healthcare workers and the evolving demands on the nursing profession in managing both the acute and chronic phases of the pandemic. These discussions underscored the multifaceted nature of long Covid, emphasising the need for comprehensive intervention strategies that address both health and social dimensions [31].

## 6 Causal Loop Mapping

### 6.1 In‐Person

**Figure 1:**
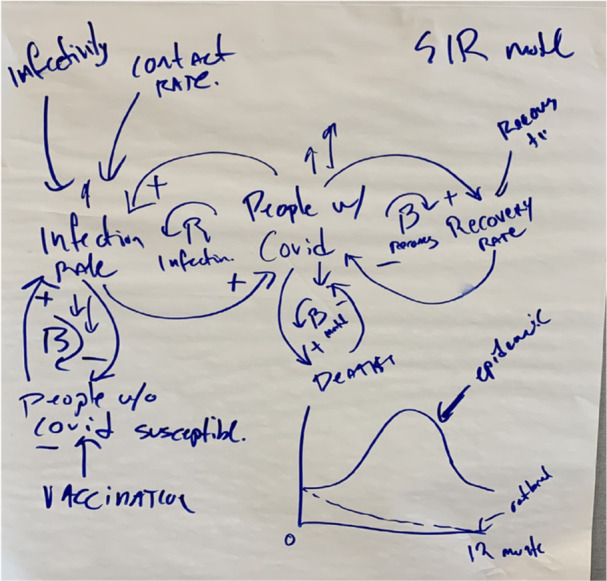
Requested reference mode from the in‐person workshop focused on Covid.

As an introduction to causal loop diagramming, the research team chose a reference mode of the chicken and egg. The chicken and egg reference mode is a standard starting point in GMB [32]. This was quickly rejected by the study participants, who instead preferred to draw inspiration from the behaviour over time graphs created earlier in the session, aligning with how participatory approaches allow stakeholders to shift the focus of the modelling effort and draw upon previously generated insights such as those from trends over time graph [33]. Adhering to the participants' request, we pivoted to a CLD of a variation of the susceptibility of infectious disease (Figure 1). Participants continued to work in their assigned groups and began drawing CLDs.

### 6.2 In‐Person CLD Insights

❖ Group 1—This group highlighted the goal of enabling people living without Covid to maintain their workout routines. They insightfully integrated the presence of the virus into the model.
❖ Group 2—This group identified a positive correlation between financial stability and the ease of managing medication regimens. They also noted that improved recovery can reduce symptoms such as shortness of breath.
❖ Group 3—Focused on the supply chain and basic needs, this group mapped out essential elements like shelter, clothing, counselling and medication. They also included the role of community activists in engaging and supporting individuals.
❖ Group 4—This group examined the impact of mandatory vaccination on civil liberties. Their second map concentrated on strategies to reduce prolonged hospital stays following infection.

**Figure 2:**
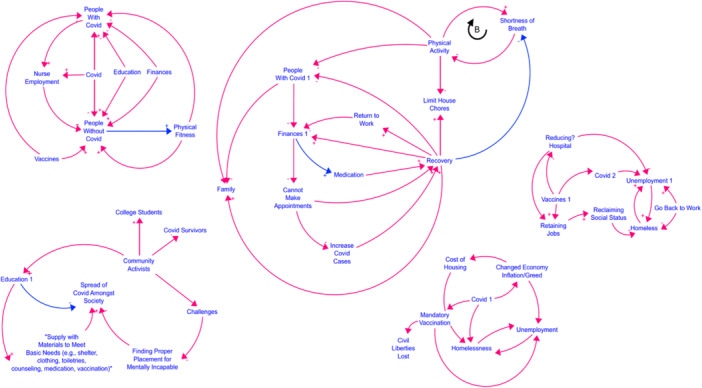
Combined causal loop diagram from the in‐person groups.

Following an online meeting with in‐person workshop participants, the research team synthesised the CLDs from all four groups into a comprehensive, unified diagram (Figure 2). Participants responded positively to this merged model, appreciating the holistic representation of their experiences [34]. A critical insight identified by the participants was the need for in‐person workshop sessions. While participants were generally open to online workshops, they expressed concerns about the potential inability to match the intensity, interaction and camaraderie of in‐person sessions. Face‐to‐face interaction was highlighted as a key factor in ensuring full engagement in workshop activities, a point well‐documented in the literature. Participants also shared that the workshop prompted them to reassess how their symptoms, such as exhaustion while walking, significantly impact their quality of life.

### 6.3 Online

**Figure 3:**
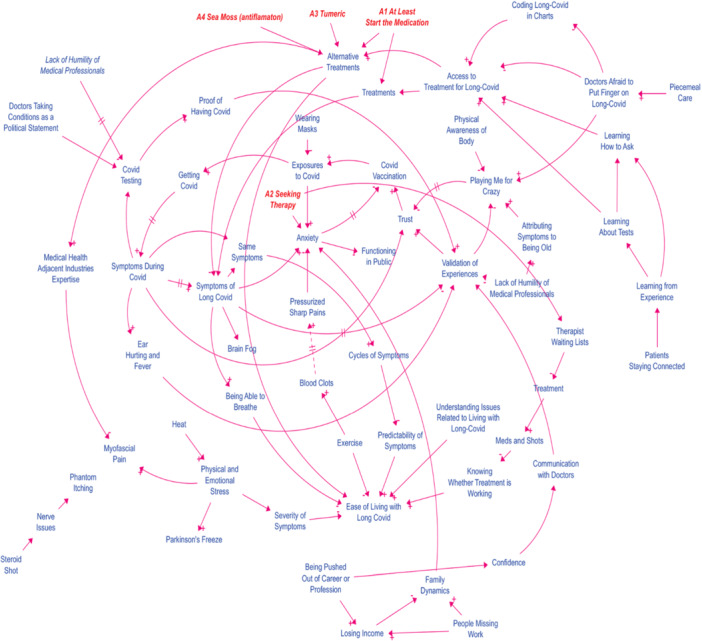
Updated causal loop diagram with action ideas from online workshop.

The CLD developed during the online workshop provides an illustrative representation of the complex interactions between various factors influencing the experience and management of Covid‐19 and long Covid. The final CLD illustrates the dynamic interplay of factors influencing the long Covid experience (Figure 3). A key reinforcing loop shows how validation of experiences enhances trust, which improves functioning in public, creating a virtuous cycle. In contrast, a balancing loop demonstrates how rising symptoms during Covid leads to more Covid testing, a necessary intervention to manage the condition. The model underscores the critical role of psychosocial factors like trust and validation in driving overall system behaviour.

The diagram distinguishes between positive and negative feedback loops, which are critical in understanding the system's behaviour over time. Reinforcing loops, identified as positive feedback loops, amplify changes within the system. For instance, the variable ‘Validation of Experiences’ positively influences ‘Trust’, which subsequently enhances ‘Functioning in Public’, further reinforcing ‘Validation of Experiences’. This loop illustrates how the acknowledgement of patient experiences can create a cycle of increased trust and social functioning.

Conversely, the diagram also includes balancing loops, or negative feedback loops, which serve to counteract changes and maintain stability within the system. An example of a balancing loop is the relationship between ‘Symptoms During Covid’ and ‘Covid Testing’. As symptoms increase, the demand for testing rises, which, in turn, contributes to reducing the symptoms through appropriate treatment and management. This loop illustrates the role of diagnostic interventions in mitigating the severity of symptoms and controlling the spread of the virus.

The interplay between reinforcing and balancing loops in the CLD highlights the dynamic nature of the healthcare system's response to Covid‐19 and long Covid. The reinforcing loops underscore the importance of factors that can enhance patient outcomes through positive feedback mechanisms, while the balancing loops emphasise the role of interventions in stabilising the system and preventing adverse outcomes. The inclusion of variables such as ‘Trust’ and ‘Validation of Experiences’ also points to the critical role of psychosocial factors in the management of long Covid, suggesting that, beyond clinical interventions, there is a need for healthcare systems to address the psychological and social dimensions of patient care.

Overall, the CLD provides valuable insights into the interconnectedness of various factors that influence the patient experience with Covid‐19 and long Covid. By identifying key variables and understanding the feedback loops that drive the system, this model can inform more effective and holistic approaches to treatment and patient management. It is important to stress that these initial workshops were to engage communities and persons living with long Covid early in the process of developing a method and assessing the feasibility of the approach, not developing a comprehensive or validated account of long Covid and symptoms. Moreover, while member checking as a form of qualitative validation is concurrent within a group model‐building workshop, this generally only applies to variables and causal links, not feedback loops identified in a CLD [35]. Additional research to develop a validated CLD could focus on replicating the workshops across more groups, member checking identified loops with lived experience supported by quotations, and comparisons against published literature. Further research could also build on this model to explore specific interventions that might optimise the balance between reinforcing and balancing loops, ultimately improving patient outcomes in the context of long Covid.

### 6.4 Differences in In‐Person and Online Workshops

Both workshops demonstrated distinct strengths in fostering productive outcomes. The in‐person workshop notably facilitated a deeper sense of camaraderie among participants, which enhanced interpersonal connections and collaborative engagement. This aligns with the recognised benefits of face‐to‐face interaction in methods like GMB, which is considered essential for exploring a shared view together and can lead to greater engagement. In contrast, the online workshop yielded a more comprehensive CLD. This increased comprehensiveness can be attributed to the study team's proactive role in using Stella Architect software to create the diagrams, thereby accelerating the rate of model development and refinement. These contrasting yet complementary strengths underscore the value of both in‐person and online modalities in advancing our understanding and management of long Covid [31].

## 7 Discussion

This pilot study demonstrates that CBSD is a vital participatory framework for advancing health equity in African American communities grappling with long Covid. Through collaborative workshops, this study provided a unique platform for individuals with lived experience to articulate the systemic dynamics of their condition. Centring community perspectives in the model‐building process offers a nuanced understanding of long Covid that traditional approaches may miss. Embedding CBSD early in the research process, before interventions are defined, holds significant promise for developing more precise and effective treatments tailored to the community's realities. As health systems confront evolving challenges, this study underscores CBSD's value as a path towards more inclusive, responsive and equitable public health research that addresses the needs of historically marginalised communities.

This study underscores the value of collaborative patient participation in health equity research. CBSD is underpinned by community‐based participatory frameworks, aiming to co‐create solutions with active community involvement and integrate with public health efforts to address complex issues [36]. Participants were not passive subjects but active contributors in shaping how the condition is understood and how interventions might be targeted. By placing individuals with lived experience at the centre of the model‐building process, CBSD aligns with current calls for greater patient inclusion in health research, as advocated by organisations like the Patient‐Centered Outcomes Research Institute. This inclusion is particularly urgent in conditions like long Covid, where emerging evidence, fragmented care pathways, and contested illness narratives intersect with broader SDoH [37].

Beyond its application to long Covid, CBSD offers a flexible and replicable methodology for use in other community health initiatives. The process fosters systems thinking at the community level, bridging gaps between experiential knowledge and public health planning. It supports the co‐production of knowledge that is both actionable and context‐specific, enhancing the likelihood that future interventions are both effective and equitable. Importantly, the collaborative mapping process also contributes to participant empowerment, trust building and engagement, which are foundational to long‐term public health impact.

Based on the workshop findings, several avenues for further research emerge. First, future studies could explore the use of CBSD as a diagnostic tool to identify leverage points for intervention before implementation, for instance, by simulating the impact of potential policy changes on healthcare access. Second, the method's capacity to adapt to various population groups suggests its potential scalability for addressing other health disparities, such as chronic disease management for individuals with diabetes, mental health inequities in underserved communities, or maternal health outcomes in rural areas. Finally, longitudinal studies could examine whether community‐designed systems models improve the relevance, uptake and sustainability of health interventions over time.

## 8 Conclusions

In conclusion, this pilot study demonstrates that CBSD is not only a powerful modelling technique but also a vital participatory framework for advancing health equity, particularly within African American communities grappling with the complexities of long Covid [38]. Through collaborative workshops, this study provided a unique platform for African American individuals with lived experience to articulate the system‐level dynamics influencing their experience of the condition [39]. By centring the perspectives of African American community members in the model‐building process, this study offers a nuanced understanding of long Covid within this population that may be missed by traditional research approaches [40]. Embedding CBSD earlier in the research process, before interventions are defined, holds significant promise for developing more precise, relevant and effective treatments and support systems tailored to the specific realities of African American individuals living with long Covid. As health systems continue to confront the evolving challenges of long Covid and other emergent conditions, this study underscores the value of CBSD as a path towards more inclusive, responsive and ultimately more equitable public health research and practice that directly addresses the needs of historically marginalised communities.

## 9 Limitations

This study has several limitations. First, the relatively small number of workshop participants (*N* = 34) limits the statistical generalisability of our findings and may not fully capture the diversity of experiences within the broader African American community with long Covid.

Second, and most notably, our workshop sample was significantly older (mean age: 67 years) than the parent observational cohort from which most participants were drawn (mean age: 41 years). This indicates a significant age‐related selection bias, likely stemming from our sequential, first‐come, first‐served recruitment method. This approach may have inadvertently favoured older, retired individuals with greater schedule flexibility over their younger, working‐age counterparts. Consequently, our systems model may place greater emphasis on challenges pertinent to older adults (e.g., managing comorbidities and navigating Medicare) while under‐representing issues critical to younger populations (e.g., workplace accommodations and childcare). Future research should employ a stratified sampling strategy to ensure a more age‐diverse sample.

Third, the CLDs are qualitative models based on participant perceptions, and as such, their predictive power has not been validated quantitatively. Fourth, despite our iterative, participant‐engaged refinement process, potential interpretive gaps between participant intent and researcher framing may still exist. Finally, the cross‐sectional nature of the workshops prevents an assessment of how participant perspectives and system dynamics may evolve over time.

Despite these limitations, this study provides an important contribution by demonstrating the utility of CBSD for integrating patient perspectives into a systems‐level understanding of long Covid, thereby laying a crucial foundation for the design of equitable interventions.

## Author Contributions

**Chad R. Evans:** conceptualisation, investigation, writing – original draft, methodology, writing – review and editing, data curation, investigation, formal analysis. **Melvin R. Echols:** conceptualisation, writing – original draft, methodology, writing – review and editing, supervision. **D. Martin Jr:** conceptualisation, writing – original draft, methodology, writing – review and editing, validation, supervision. **H. A. Taylor:** conceptualisation, investigation, writing – original draft, methodology, writing – review and editing, validation, supervision. **James A. Washington:** conceptualisation, investigation, writing – original draft, methodology, writing – review and editing. **Olusola Gbinigie:** writing – original draft, writing – review and editing, project administration. **Anne H. Gaglioti:** conceptualisation, investigation, writing‐original draft, methodology, writing – review and editing. **Wendi Wright:** writing – review and editing. **Peter Hovmand:** conceptualisation, investigation, writing – original draft, methodology, writing – review and editing, validation, supervision, formal analysis.

## Ethics Statement

The observational study and the subsequent CBSD workshops were approved by the Morehouse School of Medicine Institutional Review Board. Written informed consent was obtained from all participants before their enrollment in the original observational study, and separate informed consent was obtained from all 29 participants who agreed to participate in the CBSD workshops after receiving a full explanation of the workshop's purpose and procedures.

## Conflicts of Interest

The authors declare no conflicts of interest.

## Data Availability

The data that support the findings of this study are available on request from the corresponding author. The data are not publicly available due to privacy or ethical restrictions.
